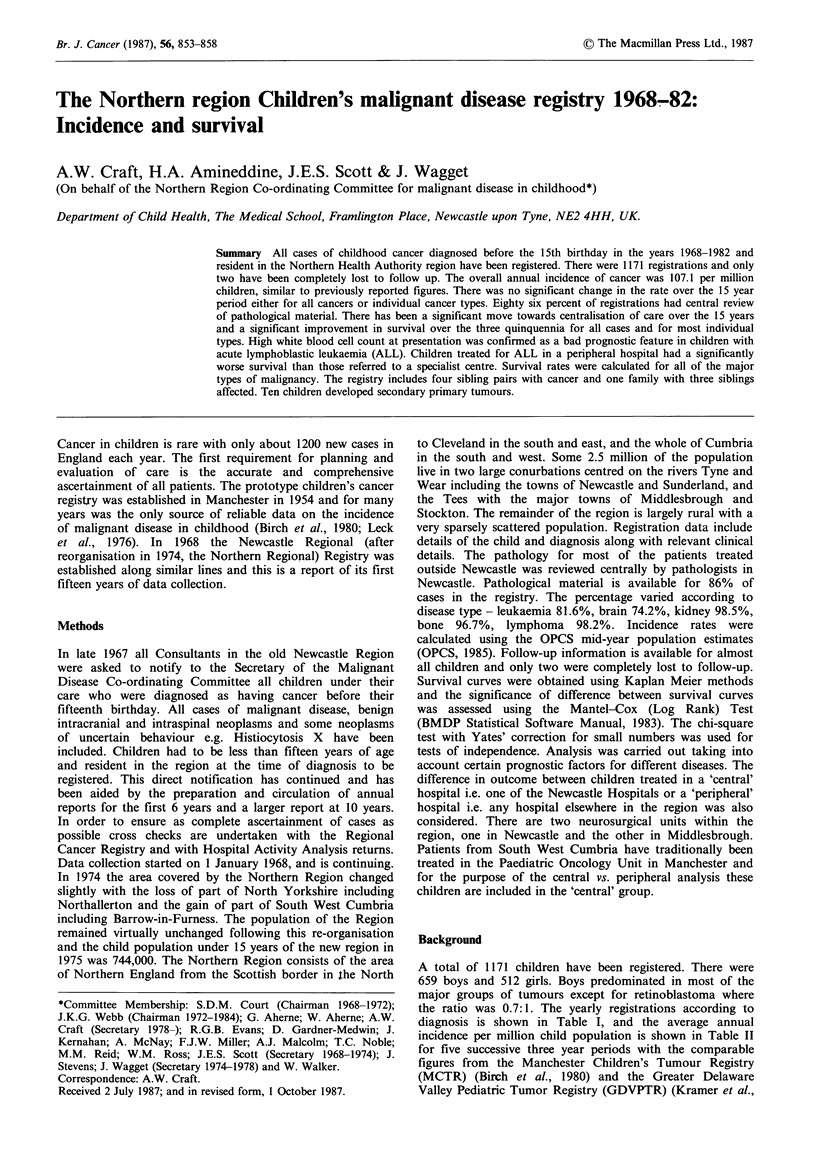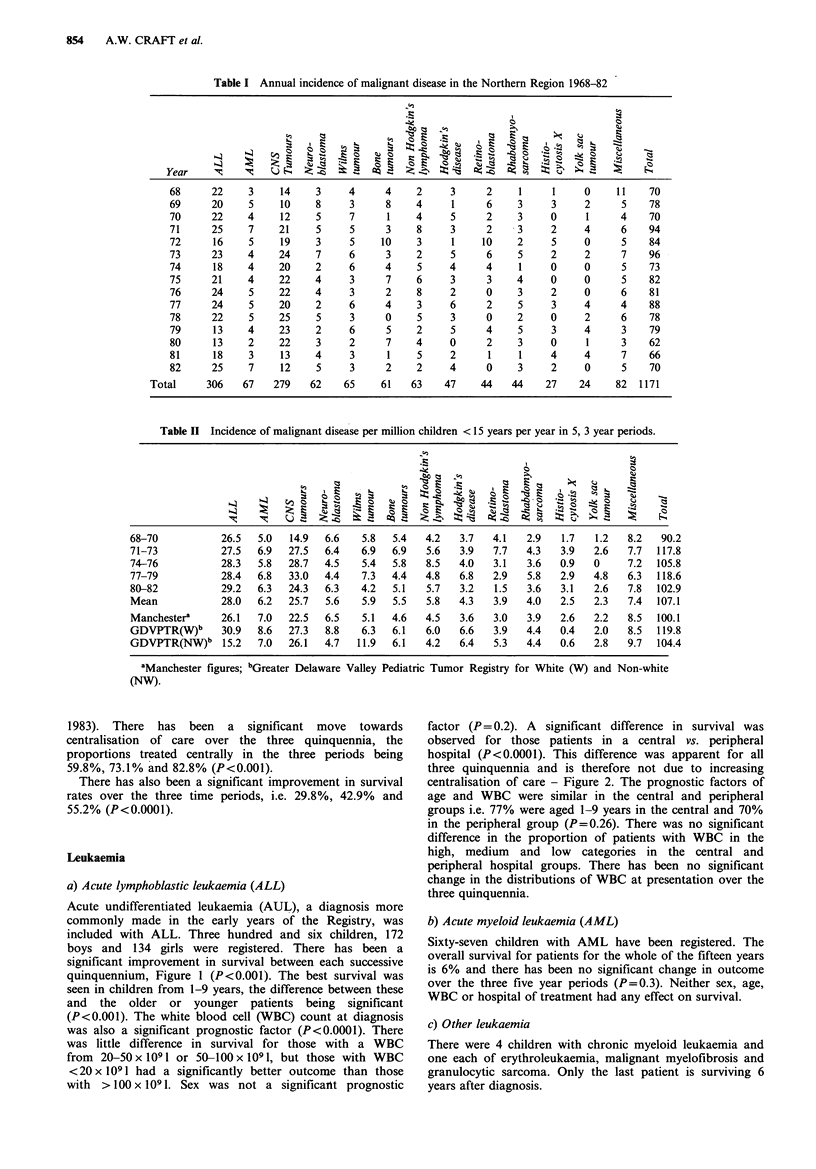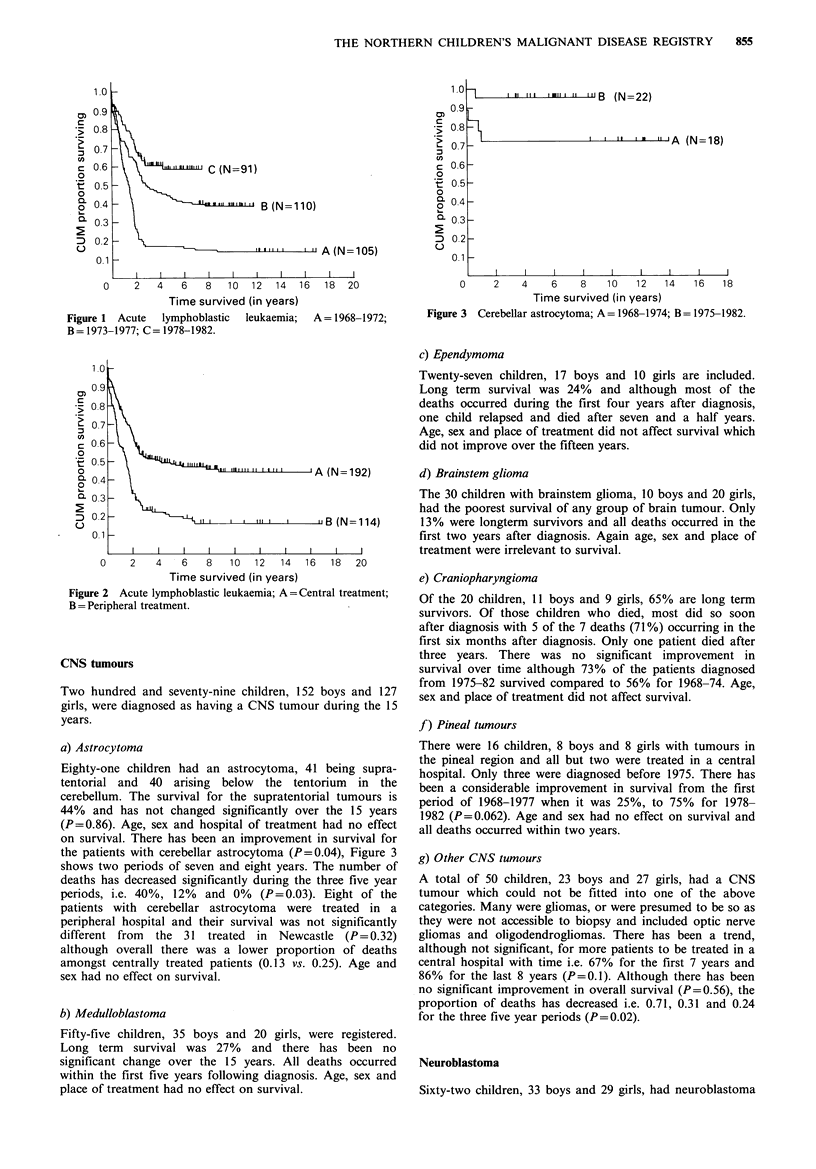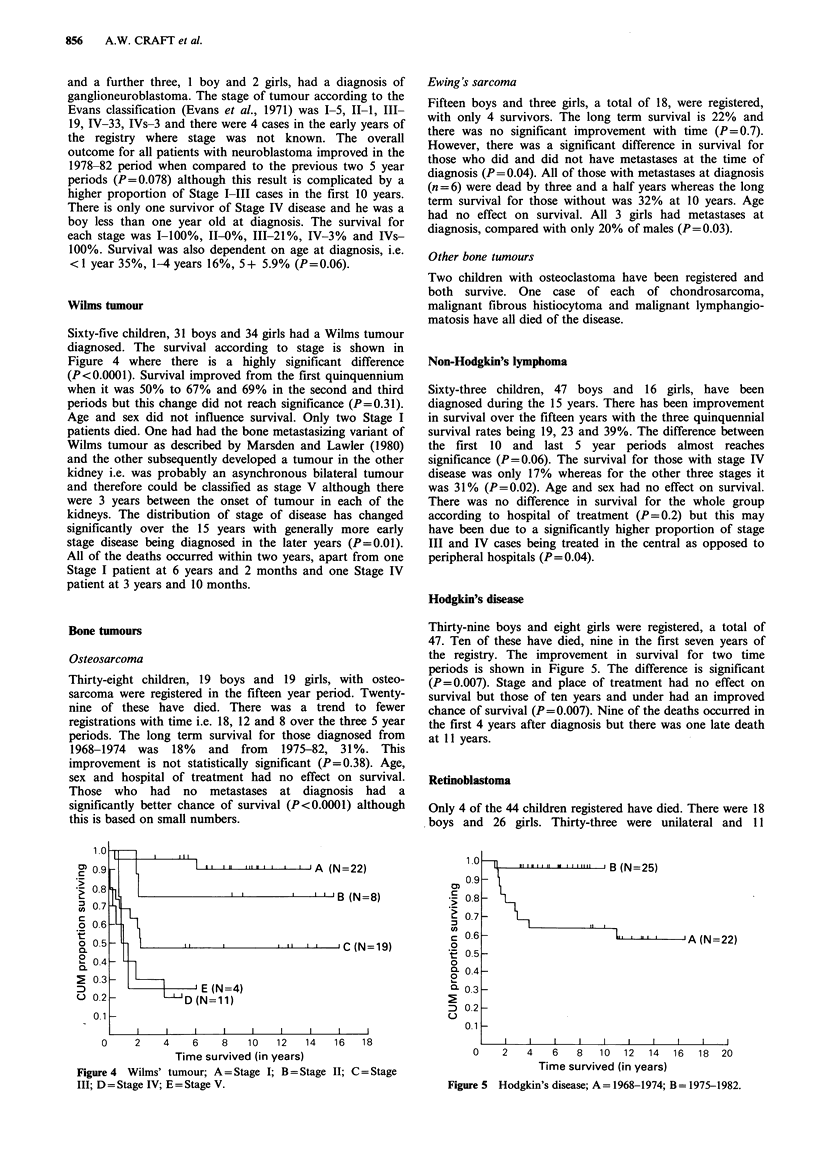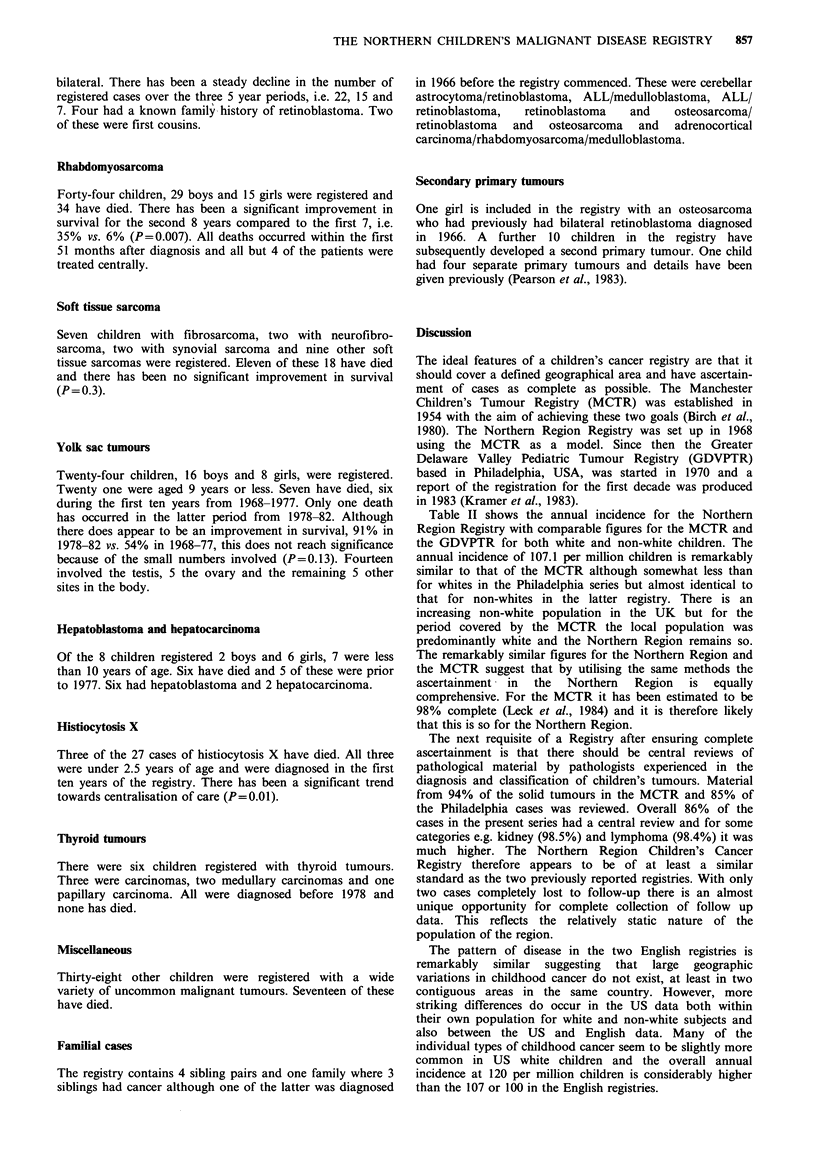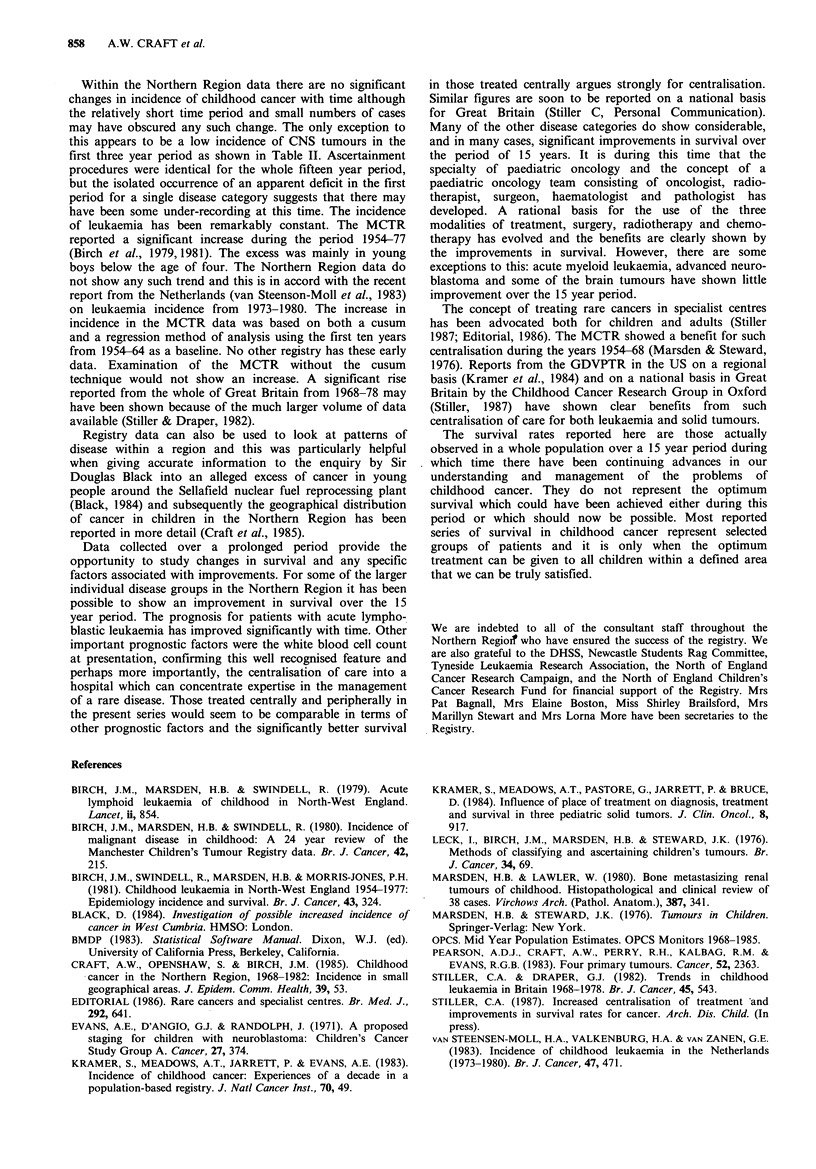# The Northern region Children's malignant disease registry 1968-82: incidence and survival.

**DOI:** 10.1038/bjc.1987.305

**Published:** 1987-12

**Authors:** A. W. Craft, H. A. Amineddine, J. E. Scott, J. Wagget

**Affiliations:** Department of Child Health, The Medical School, Newcastle upon Tyne, UK.

## Abstract

All cases of childhood cancer diagnosed before the 15th birthday in the years 1968-1982 and resident in the Northern Health Authority region have been registered. There were 1171 registrations and only two have been completely lost to follow up. The overall annual incidence of cancer was 107.1 per million children, similar to previously reported figures. There was no significant change in the rate over the 15 year period either for all cancers or individual cancer types. Eighty six percent of registrations had central review of pathological material. There has been a significant move towards centralisation of care over the 15 years and a significant improvement in survival over the three quinquennia for all cases and for most individual types. High white blood cell count at presentation was confirmed as a bad prognostic feature in children with acute lymphoblastic leukaemia (ALL). Children treated for ALL in a peripheral hospital had a significantly worse survival than those referred to a specialist centre. Survival rates were calculated for all of the major types of malignancy. The registry includes four sibling pairs with cancer and one family with three siblings affected. Ten children developed secondary primary tumours.


*Br. J. Cancer* (1987), **56**, 853–858 © The Macmillan Press Ltd., 1987

### The Northern region Children's malignant disease registry 1968–82: Incidence and survival

A.W. Craft, H.A. Amineddine, J.E.S. Scott & J. Wagget
(On behalf of the Northern Region Co-ordinating Committee for malignant disease in childhood*)

*Department of Child Health, The Medical School, Framlington Place, Newcastle upon Tyne, NE2 4HH, UK.*

**Summary** All cases of childhood cancer diagnosed before the 15th birthday in the years 1968–1982 and resident in the Northern Health Authority region have been registered. There were 1171 registrations and only two have been completely lost to follow up. The overall annual incidence of cancer was 107.1 per million children, similar to previously reported figures. There was no significant change in the rate over the 15 year period either for all cancers or individual cancer types. Eighty six percent of registrations had central review of pathological material. There has been a significant move towards centralisation of care over the 15 years and a significant improvement in survival over the three quinquennia for all cases and for most individual types. High white blood cell count at presentation was confirmed as a bad prognostic feature in children with acute lymphoblastic leukaemia (ALL). Children treated for ALL in a peripheral hospital had a significantly worse survival than those referred to a specialist centre. Survival rates were calculated for all of the major types of malignancy. The registry includes four sibling pairs with cancer and one family with three siblings affected. Ten children developed secondary primary tumours.

Cancer in children is rare with only about 1200 new cases in England each year. The first requirement for planning and evaluation of care is the accurate and comprehensive ascertainment of all patients. The prototype children's cancer registry was established in Manchester in 1954 and for many years was the only source of reliable data on the incidence of malignant disease in childhood (Birch *et al.*, 1980; Leck *et al.*, 1976). In 1968 the Newcastle Regional (after reorganisation in 1974, the Northern Regional) Registry was established along similar lines and this is a report of its first fifteen years of data collection.

### Methods

In late 1967 all Consultants in the old Newcastle Region were asked to notify to the Secretary of the Malignant Disease Co-ordinating Committee all children under their care who were diagnosed as having cancer before their fifteenth birthday. All cases of malignant disease, benign intracranial and intraspinal neoplasms and some neoplasms of uncertain behaviour e.g. Histiocytosis X have been included. Children had to be less than fifteen years of age and resident in the region at the time of diagnosis to be registered. This direct notification has continued and has been aided by the preparation and circulation of annual reports for the first 6 years and a larger report at 10 years. In order to ensure as complete ascertainment of cases as possible cross checks are undertaken with the Regional Cancer Registry and with Hospital Activity Analysis returns. Data collection started on 1 January 1968, and is continuing. In 1974 the area covered by the Northern Region changed slightly with the loss of part of North Yorkshire including Northallerton and the gain of part of South West Cumbria including Barrow-in-Furness. The population of the Region remained virtually unchanged following this re-organisation and the child population under 15 years of the new region in 1975 was 744,000. The Northern Region consists of the area of Northern England from the Scottish border in the North to Cleveland in the south and east, and the whole of Cumbria in the south and west. Some 2.5 million of the population live in two large conurbations centred on the rivers Tyne and Wear including the towns of Newcastle and Sunderland, and the Tees with the major towns of Middlesbrough and Stockton. The remainder of the region is largely rural with a very sparsely scattered population. Registration data include details of the child and diagnosis along with relevant clinical details. The pathology for most of the patients treated outside Newcastle was reviewed centrally by pathologists in Newcastle. Pathological material is available for 86% of cases in the registry. The percentage varied according to disease type – leukaemia 81.6%, brain 74.2%, kidney 98.5%, bone 96.7%, lymphoma 98.2%. Incidence rates were calculated using the OPCS mid-year population estimates (OPCS, 1985). Follow-up information is available for almost all children and only two were completely lost to follow-up. Survival curves were obtained using Kaplan Meier methods and the significance of difference between survival curves was assessed using the Mantel–Cox (Log Rank) Test (BMDP Statistical Software Manual, 1983). The chi-square test with Yates' correction for small numbers was used for tests of independence. Analysis was carried out taking into account certain prognostic factors for different diseases. The difference in outcome between children treated in a 'central' hospital i.e. one of the Newcastle Hospitals or a 'peripheral' hospital i.e. any hospital elsewhere in the region was also considered. There are two neurosurgical units within the region, one in Newcastle and the other in Middlesbrough. Patients from South West Cumbria have traditionally been treated in the Paediatric Oncology Unit in Manchester and for the purpose of the central *vs.* peripheral analysis these children are included in the 'central' group.

### Background

A total of 1171 children have been registered. There were 659 boys and 512 girls. Boys predominated in most of the major groups of tumours except for retinoblastoma where the ratio was 0.7:1. The yearly registrations according to diagnosis is shown in Table I, and the average annual incidence per million child population is shown in Table II for five successive three year periods with the comparable figures from the Manchester Children's Tumour Registry (MCTR) (Birch *et al.*, 1980) and the Greater Delaware Valley Pediatric Tumor Registry (GDVPTR) (Kramer *et al.*,

*Committee Membership: S.D.M. Court (Chairman 1968–1972); J.K.G. Webb (Chairman 1972–1984); G. Aherne; W. Aherne; A.W. Craft (Secretary 1978–); R.G.B. Evans; D. Gardner-Medwin; J. Kernahan; A. McNay; F.J.W. Miller; A.J. Malcolm; T.C. Noble; M.M. Reid; W.M. Ross; J.E.S. Scott (Secretary 1968–1974); J. Stevens; J. Wagget (Secretary 1974–1978) and W. Walker.
Correspondence: A.W. Craft.
Received 2 July 1987; and in revised form, 1 October 1987.

**Table I** Annual incidence of malignant disease in the Northern Region 1968–82

| *Year* | *ALL* | *AML* | *CNS Tumours* | *Neuro-blastoma* | *Wilms tumour* | *Bone tumours* | *Non Hodgkin's lymphoma* | *Hodgkin's disease* | *Retino-blastoma* | *Rhabdomyo-sarcoma* | *Histio-cytosis X* | *Yolk sac tumour* | *Miscellaneous* | *Total* |
|---|---|---|---|---|---|---|---|---|---|---|---|---|---|---|
| 68 | 22 | 3 | 14 | 3 | 4 | 4 | 2 | 3 | 2 | 1 | 1 | 0 | 11 | 70 |
| 69 | 20 | 5 | 10 | 8 | 3 | 8 | 4 | 1 | 6 | 3 | 3 | 2 | 5 | 78 |
| 70 | 22 | 4 | 12 | 5 | 7 | 1 | 4 | 5 | 2 | 3 | 0 | 1 | 4 | 70 |
| 71 | 25 | 7 | 21 | 5 | 5 | 3 | 8 | 3 | 2 | 3 | 2 | 4 | 6 | 94 |
| 72 | 16 | 5 | 19 | 3 | 5 | 10 | 3 | 1 | 10 | 2 | 5 | 0 | 5 | 84 |
| 73 | 23 | 4 | 24 | 7 | 6 | 3 | 2 | 5 | 6 | 5 | 2 | 2 | 7 | 96 |
| 74 | 18 | 4 | 20 | 2 | 6 | 4 | 5 | 4 | 4 | 1 | 0 | 0 | 5 | 73 |
| 75 | 21 | 4 | 22 | 4 | 3 | 7 | 6 | 3 | 3 | 4 | 0 | 0 | 5 | 82 |
| 76 | 24 | 5 | 22 | 4 | 3 | 2 | 8 | 2 | 0 | 3 | 2 | 0 | 6 | 81 |
| 77 | 24 | 5 | 20 | 2 | 6 | 4 | 3 | 6 | 2 | 5 | 3 | 4 | 4 | 88 |
| 78 | 22 | 5 | 25 | 5 | 3 | 0 | 5 | 3 | 0 | 2 | 0 | 2 | 6 | 78 |
| 79 | 13 | 4 | 23 | 2 | 6 | 5 | 2 | 5 | 4 | 5 | 3 | 4 | 3 | 79 |
| 80 | 13 | 2 | 22 | 3 | 2 | 7 | 4 | 0 | 2 | 3 | 0 | 1 | 3 | 62 |
| 81 | 18 | 3 | 13 | 4 | 3 | 1 | 5 | 2 | 1 | 1 | 4 | 4 | 7 | 66 |
| 82 | 25 | 7 | 12 | 5 | 3 | 2 | 2 | 4 | 0 | 3 | 2 | 0 | 5 | 70 |
| Total | 306 | 67 | 279 | 62 | 65 | 61 | 63 | 47 | 44 | 44 | 27 | 24 | 82 | 1171 |

**Table II** Incidence of malignant disease per million children <15 years per year in 5, 3 year periods.

| | *ALL* | *AML* | *CNS tumours* | *Neuro-blastoma* | *Wilms tumour* | *Bone tumours* | *Non Hodgkin's lymphoma* | *Hodgkin's disease* | *Retino-blastoma* | *Rhabdomyo-sarcoma* | *Histio-cytosis X* | *Yolk sac tumour* | *Miscellaneous* | *Total* |
|---|---|---|---|---|---|---|---|---|---|---|---|---|---|---|
| 68–70 | 26.5 | 5.0 | 14.9 | 6.6 | 5.8 | 5.4 | 4.2 | 3.7 | 4.1 | 2.9 | 1.7 | 1.2 | 8.2 | 90.2 |
| 71–73 | 27.5 | 6.9 | 27.5 | 6.4 | 6.9 | 6.9 | 5.6 | 3.9 | 7.7 | 4.3 | 3.9 | 2.6 | 7.7 | 117.8 |
| 74–76 | 28.3 | 5.8 | 28.7 | 4.5 | 5.4 | 5.8 | 8.5 | 4.0 | 3.1 | 3.6 | 0.9 | 0 | 7.2 | 105.8 |
| 77–79 | 28.4 | 6.8 | 33.0 | 4.4 | 7.3 | 4.4 | 4.8 | 6.8 | 2.9 | 5.8 | 2.9 | 4.8 | 6.3 | 118.6 |
| 80–82 | 29.2 | 6.3 | 24.3 | 6.3 | 4.2 | 5.1 | 5.7 | 3.2 | 1.5 | 3.6 | 3.1 | 2.6 | 7.8 | 102.9 |
| Mean | 28.0 | 6.2 | 25.7 | 5.6 | 5.9 | 5.5 | 5.8 | 4.3 | 3.9 | 4.0 | 2.5 | 2.3 | 7.4 | 107.1 |
| Manchester[a] | 26.1 | 7.0 | 22.5 | 6.5 | 5.1 | 4.6 | 4.5 | 3.6 | 3.0 | 3.9 | 2.6 | 2.2 | 8.5 | 100.1 |
| GDVPTR(W)[b] | 30.9 | 8.6 | 27.3 | 8.8 | 6.3 | 6.1 | 6.0 | 6.6 | 3.9 | 4.4 | 0.4 | 2.0 | 8.5 | 119.8 |
| GDVPTR(NW)[b] | 15.2 | 7.0 | 26.1 | 4.7 | 11.9 | 6.1 | 4.2 | 6.4 | 5.3 | 4.4 | 0.6 | 2.8 | 9.7 | 104.4 |

[a]Manchester figures; [b]Greater Delaware Valley Pediatric Tumor Registry for White (W) and Non-white (NW).

1983). There has been a significant move towards centralisation of care over the three quinquennia, the proportions treated centrally in the three periods being 59.8%, 73.1% and 82.8% ($P<0.001$).

There has also been a significant improvement in survival rates over the three time periods, i.e. 29.8%, 42.9% and 55.2% ($P<0.0001$).

### Leukaemia

### *a) Acute lymphoblastic leukaemia (ALL)*

Acute undifferentiated leukaemia (AUL), a diagnosis more commonly made in the early years of the Registry, was included with ALL. Three hundred and six children, 172 boys and 134 girls were registered. There has been a significant improvement in survival between each successive quinquennium, Figure 1 ($P<0.001$). The best survival was seen in children from 1–9 years, the difference between these and the older or younger patients being significant ($P<0.001$). The white blood cell (WBC) count at diagnosis was also a significant prognostic factor ($P<0.0001$). There was little difference in survival for those with a WBC from $20–50\times10^9$ l or $50–100\times10^9$ l, but those with WBC $<20\times10^9$ l had a significantly better outcome than those with $>100\times10^9$ l. Sex was not a significant prognostic factor ($P=0.2$). A significant difference in survival was observed for those patients in a central *vs.* peripheral hospital ($P<0.0001$). This difference was apparent for all three quinquennia and is therefore not due to increasing centralisation of care – Figure 2. The prognostic factors of age and WBC were similar in the central and peripheral groups i.e. 77% were aged 1–9 years in the central and 70% in the peripheral group ($P=0.26$). There was no significant difference in the proportion of patients with WBC in the high, medium and low categories in the central and peripheral hospital groups. There has been no significant change in the distributions of WBC at presentation over the three quinquennia.

### *b) Acute myeloid leukaemia (AML)*

Sixty-seven children with AML have been registered. The overall survival for patients for the whole of the fifteen years is 6% and there has been no significant change in outcome over the three five year periods ($P=0.3$). Neither sex, age, WBC or hospital of treatment had any effect on survival.

### *c) Other leukaemia*

There were 4 children with chronic myeloid leukaemia and one each of erythroleukaemia, malignant myelofibrosis and granulocytic sarcoma. Only the last patient is surviving 6 years after diagnosis.

**Figure 1** Acute lymphoblastic leukaemia; A = 1968–1972; B = 1973–1977; C = 1978–1982.

**Figure 2** Acute lymphoblastic leukaemia; A = Central treatment; B = Peripheral treatment.

### CNS tumours

Two hundred and seventy-nine children, 152 boys and 127 girls, were diagnosed as having a CNS tumour during the 15 years.

### *a) Astrocytoma*

Eighty-one children had an astrocytoma, 41 being supratentorial and 40 arising below the tentorium in the cerebellum. The survival for the supratentorial tumours is 44% and has not changed significantly over the 15 years ($P = 0.86$). Age, sex and hospital of treatment had no effect on survival. There has been an improvement in survival for the patients with cerebellar astrocytoma ($P = 0.04$), Figure 3 shows two periods of seven and eight years. The number of deaths has decreased significantly during the three five year periods, i.e. 40%, 12% and 0% ($P = 0.03$). Eight of the patients with cerebellar astrocytoma were treated in a peripheral hospital and their survival was not significantly different from the 31 treated in Newcastle ($P = 0.32$) although overall there was a lower proportion of deaths amongst centrally treated patients (0.13 *vs.* 0.25). Age and sex had no effect on survival.

### *b) Medulloblastoma*

Fifty-five children, 35 boys and 20 girls, were registered. Long term survival was 27% and there has been no significant change over the 15 years. All deaths occurred within the first five years following diagnosis. Age, sex and place of treatment had no effect on survival.

**Figure 3** Cerebellar astrocytoma; A = 1968–1974; B = 1975–1982.

### *c) Ependymoma*

Twenty-seven children, 17 boys and 10 girls are included. Long term survival was 24% and although most of the deaths occurred during the first four years after diagnosis, one child relapsed and died after seven and a half years. Age, sex and place of treatment did not affect survival which did not improve over the fifteen years.

### *d) Brainstem glioma*

The 30 children with brainstem glioma, 10 boys and 20 girls, had the poorest survival of any group of brain tumour. Only 13% were longterm survivors and all deaths occurred in the first two years after diagnosis. Again age, sex and place of treatment were irrelevant to survival.

### *e) Craniopharyngioma*

Of the 20 children, 11 boys and 9 girls, 65% are long term survivors. Of those children who died, most did so soon after diagnosis with 5 of the 7 deaths (71%) occurring in the first six months after diagnosis. Only one patient died after three years. There was no significant improvement in survival over time although 73% of the patients diagnosed from 1975–82 survived compared to 56% for 1968–74. Age, sex and place of treatment did not affect survival.

### *f) Pineal tumours*

There were 16 children, 8 boys and 8 girls with tumours in the pineal region and all but two were treated in a central hospital. Only three were diagnosed before 1975. There has been a considerable improvement in survival from the first period of 1968–1977 when it was 25%, to 75% for 1978–1982 ($P = 0.062$). Age and sex had no effect on survival and all deaths occurred within two years.

### *g) Other CNS tumours*

A total of 50 children, 23 boys and 27 girls, had a CNS tumour which could not be fitted into one of the above categories. Many were gliomas, or were presumed to be so as they were not accessible to biopsy and included optic nerve gliomas and oligodendrogliomas. There has been a trend, although not significant, for more patients to be treated in a central hospital with time i.e. 67% for the first 7 years and 86% for the last 8 years ($P = 0.1$). Although there has been no significant improvement in overall survival ($P = 0.56$), the proportion of deaths has decreased i.e. 0.71, 0.31 and 0.24 for the three five year periods ($P = 0.02$).

### Neuroblastoma

Sixty-two children, 33 boys and 29 girls, had neuroblastoma and a further three, 1 boy and 2 girls, had a diagnosis of ganglioneuroblastoma. The stage of tumour according to the Evans classification (Evans *et al.*, 1971) was I–5, II–1, III–19, IV–33, IVs–3 and there were 4 cases in the early years of the registry where stage was not known. The overall outcome for all patients with neuroblastoma improved in the 1978–82 period when compared to the previous two 5 year periods ($P = 0.078$) although this result is complicated by a higher proportion of Stage I–III cases in the first 10 years. There is only one survivor of Stage IV disease and he was a boy less than one year old at diagnosis. The survival for each stage was I–100%, II–0%, III–21%, IV–3% and IVs–100%. Survival was also dependent on age at diagnosis, i.e. <1 year 35%, 1–4 years 16%, 5+ 5.9% ($P = 0.06$).

### Wilms tumour

Sixty-five children, 31 boys and 34 girls had a Wilms tumour diagnosed. The survival according to stage is shown in Figure 4 where there is a highly significant difference ($P < 0.0001$). Survival improved from the first quinquennium when it was 50% to 67% and 69% in the second and third periods but this change did not reach significance ($P = 0.31$). Age and sex did not influence survival. Only two Stage I patients died. One had had the bone metastasizing variant of Wilms tumour as described by Marsden and Lawler (1980) and the other subsequently developed a tumour in the other kidney i.e. was probably an asynchronous bilateral tumour and therefore could be classified as stage V although there were 3 years between the onset of tumour in each of the kidneys. The distribution of stage of disease has changed significantly over the 15 years with generally more early stage disease being diagnosed in the later years ($P = 0.01$). All of the deaths occurred within two years, apart from one Stage I patient at 6 years and 2 months and one Stage IV patient at 3 years and 10 months.

### Bone tumours

### *Osteosarcoma*

Thirty-eight children, 19 boys and 19 girls, with osteosarcoma were registered in the fifteen year period. Twenty-nine of these have died. There was a trend to fewer registrations with time i.e. 18, 12 and 8 over the three 5 year periods. The long term survival for those diagnosed from 1968–1974 was 18% and from 1975–82, 31%. This improvement is not statistically significant ($P = 0.38$). Age, sex and hospital of treatment had no effect on survival. Those who had no metastases at diagnosis had a significantly better chance of survival ($P < 0.0001$) although this is based on small numbers.

**Figure 4** Wilms' tumour; A = Stage I; B = Stage II; C = Stage III; D = Stage IV; E = Stage V.

### *Ewing's sarcoma*

Fifteen boys and three girls, a total of 18, were registered, with only 4 survivors. The long term survival is 22% and there was no significant improvement with time ($P = 0.7$). However, there was a significant difference in survival for those who did and did not have metastases at the time of diagnosis ($P = 0.04$). All of those with metastases at diagnosis ($n = 6$) were dead by three and a half years whereas the long term survival for those without was 32% at 10 years. Age had no effect on survival. All 3 girls had metastases at diagnosis, compared with only 20% of males ($P = 0.03$).

### *Other bone tumours*

Two children with osteoclastoma have been registered and both survive. One case of each of chondrosarcoma, malignant fibrous histiocytoma and malignant lymphangiomatosis have all died of the disease.

### Non-Hodgkin's lymphoma

Sixty-three children, 47 boys and 16 girls, have been diagnosed during the 15 years. There has been improvement in survival over the fifteen years with the three quinquennial survival rates being 19, 23 and 39%. The difference between the first 10 and last 5 year periods almost reaches significance ($P = 0.06$). The survival for those with stage IV disease was only 17% whereas for the other three stages it was 31% ($P = 0.02$). Age and sex had no effect on survival. There was no difference in survival for the whole group according to hospital of treatment ($P = 0.2$) but this may have been due to a significantly higher proportion of stage III and IV cases being treated in the central as opposed to peripheral hospitals ($P = 0.04$).

### Hodgkin's disease

Thirty-nine boys and eight girls were registered, a total of 47. Ten of these have died, nine in the first seven years of the registry. The improvement in survival for two time periods is shown in Figure 5. The difference is significant ($P = 0.007$). Stage and place of treatment had no effect on survival but those of ten years and under had an improved chance of survival ($P = 0.007$). Nine of the deaths occurred in the first 4 years after diagnosis but there was one late death at 11 years.

### Retinoblastoma

Only 4 of the 44 children registered have died. There were 18 boys and 26 girls. Thirty-three were unilateral and 11

**Figure 5** Hodgkin's disease; A = 1968–1974; B = 1975–1982.

bilateral. There has been a steady decline in the number of registered cases over the three 5 year periods, i.e. 22, 15 and 7. Four had a known family history of retinoblastoma. Two of these were first cousins.

**Rhabdomyosarcoma**

Forty-four children, 29 boys and 15 girls were registered and 34 have died. There has been a significant improvement in survival for the second 8 years compared to the first 7, i.e. 35% *vs.* 6% ($P=0.007$). All deaths occurred within the first 51 months after diagnosis and all but 4 of the patients were treated centrally.

**Soft tissue sarcoma**

Seven children with fibrosarcoma, two with neurofibrosarcoma, two with synovial sarcoma and nine other soft tissue sarcomas were registered. Eleven of these 18 have died and there has been no significant improvement in survival ($P=0.3$).

**Yolk sac tumours**

Twenty-four children, 16 boys and 8 girls, were registered. Twenty one were aged 9 years or less. Seven have died, six during the first ten years from 1968–1977. Only one death has occurred in the latter period from 1978–82. Although there does appear to be an improvement in survival, 91% in 1978–82 *vs.* 54% in 1968–77, this does not reach significance because of the small numbers involved ($P=0.13$). Fourteen involved the testis, 5 the ovary and the remaining 5 other sites in the body.

**Hepatoblastoma and hepatocarcinoma**

Of the 8 children registered 2 boys and 6 girls, 7 were less than 10 years of age. Six have died and 5 of these were prior to 1977. Six had hepatoblastoma and 2 hepatocarcinoma.

**Histiocytosis X**

Three of the 27 cases of histiocytosis X have died. All three were under 2.5 years of age and were diagnosed in the first ten years of the registry. There has been a significant trend towards centralisation of care ($P=0.01$).

**Thyroid tumours**

There were six children registered with thyroid tumours. Three were carcinomas, two medullary carcinomas and one papillary carcinoma. All were diagnosed before 1978 and none has died.

**Miscellaneous**

Thirty-eight other children were registered with a wide variety of uncommon malignant tumours. Seventeen of these have died.

**Familial cases**

The registry contains 4 sibling pairs and one family where 3 siblings had cancer although one of the latter was diagnosed in 1966 before the registry commenced. These were cerebellar astrocytoma/retinoblastoma, ALL/medulloblastoma, ALL/retinoblastoma, retinoblastoma and osteosarcoma/retinoblastoma and osteosarcoma and adrenocortical carcinoma/rhabdomyosarcoma/medulloblastoma.

**Secondary primary tumours**

One girl is included in the registry with an osteosarcoma who had previously had bilateral retinoblastoma diagnosed in 1966. A further 10 children in the registry have subsequently developed a second primary tumour. One child had four separate primary tumours and details have been given previously (Pearson *et al.*, 1983).

**Discussion**

The ideal features of a children's cancer registry are that it should cover a defined geographical area and have ascertainment of cases as complete as possible. The Manchester Children's Tumour Registry (MCTR) was established in 1954 with the aim of achieving these two goals (Birch *et al.*, 1980). The Northern Region Registry was set up in 1968 using the MCTR as a model. Since then the Greater Delaware Valley Pediatric Tumour Registry (GDVPTR) based in Philadelphia, USA, was started in 1970 and a report of the registration for the first decade was produced in 1983 (Kramer *et al.*, 1983).

Table II shows the annual incidence for the Northern Region Registry with comparable figures for the MCTR and the GDVPTR for both white and non-white children. The annual incidence of 107.1 per million children is remarkably similar to that of the MCTR although somewhat less than for whites in the Philadelphia series but almost identical to that for non-whites in the latter registry. There is an increasing non-white population in the UK but for the period covered by the MCTR the local population was predominantly white and the Northern Region remains so. The remarkably similar figures for the Northern Region and the MCTR suggest that by utilising the same methods the ascertainment in the Northern Region is equally comprehensive. For the MCTR it has been estimated to be 98% complete (Leck *et al.*, 1984) and it is therefore likely that this is so for the Northern Region.

The next requisite of a Registry after ensuring complete ascertainment is that there should be central reviews of pathological material by pathologists experienced in the diagnosis and classification of children's tumours. Material from 94% of the solid tumours in the MCTR and 85% of the Philadelphia cases was reviewed. Overall 86% of the cases in the present series had a central review and for some categories e.g. kidney (98.5%) and lymphoma (98.4%) it was much higher. The Northern Region Children's Cancer Registry therefore appears to be of at least a similar standard as the two previously reported registries. With only two cases completely lost to follow-up there is an almost unique opportunity for complete collection of follow up data. This reflects the relatively static nature of the population of the region.

The pattern of disease in the two English registries is remarkably similar suggesting that large geographic variations in childhood cancer do not exist, at least in two contiguous areas in the same country. However, more striking differences do occur in the US data both within their own population for white and non-white subjects and also between the US and English data. Many of the individual types of childhood cancer seem to be slightly more common in US white children and the overall annual incidence at 120 per million children is considerably higher than the 107 or 100 in the English registries.

Within the Northern Region data there are no significant changes in incidence of childhood cancer with time although the relatively short time period and small numbers of cases may have obscured any such change. The only exception to this appears to be a low incidence of CNS tumours in the first three year period as shown in Table II. Ascertainment procedures were identical for the whole fifteen year period, but the isolated occurrence of an apparent deficit in the first period for a single disease category suggests that there may have been some under-recording at this time. The incidence of leukaemia has been remarkably constant. The MCTR reported a significant increase during the period 1954–77 (Birch *et al.*, 1979, 1981). The excess was mainly in young boys below the age of four. The Northern Region data do not show any such trend and this is in accord with the recent report from the Netherlands (van Steenson-Moll *et al.*, 1983) on leukaemia incidence from 1973–1980. The increase in incidence in the MCTR data was based on both a cusum and a regression method of analysis using the first ten years from 1954–64 as a baseline. No other registry has these early data. Examination of the MCTR without the cusum technique would not show an increase. A significant rise reported from the whole of Great Britain from 1968–78 may have been shown because of the much larger volume of data available (Stiller & Draper, 1982).

Registry data can also be used to look at patterns of disease within a region and this was particularly helpful when giving accurate information to the enquiry by Sir Douglas Black into an alleged excess of cancer in young people around the Sellafield nuclear fuel reprocessing plant (Black, 1984) and subsequently the geographical distribution of cancer in children in the Northern Region has been reported in more detail (Craft *et al.*, 1985).

Data collected over a prolonged period provide the opportunity to study changes in survival and any specific factors associated with improvements. For some of the larger individual disease groups in the Northern Region it has been possible to show an improvement in survival over the 15 year period. The prognosis for patients with acute lymphoblastic leukaemia has improved significantly with time. Other important prognostic factors were the white blood cell count at presentation, confirming this well recognised feature and perhaps more importantly, the centralisation of care into a hospital which can concentrate expertise in the management of a rare disease. Those treated centrally and peripherally in the present series would seem to be comparable in terms of other prognostic factors and the significantly better survival in those treated centrally argues strongly for centralisation. Similar figures are soon to be reported on a national basis for Great Britain (Stiller C, Personal Communication). Many of the other disease categories do show considerable, and in many cases, significant improvements in survival over the period of 15 years. It is during this time that the specialty of paediatric oncology and the concept of a paediatric oncology team consisting of oncologist, radiotherapist, surgeon, haematologist and pathologist has developed. A rational basis for the use of the three modalities of treatment, surgery, radiotherapy and chemotherapy has evolved and the benefits are clearly shown by the improvements in survival. However, there are some exceptions to this: acute myeloid leukaemia, advanced neuroblastoma and some of the brain tumours have shown little improvement over the 15 year period.

The concept of treating rare cancers in specialist centres has been advocated both for children and adults (Stiller 1987; Editorial, 1986). The MCTR showed a benefit for such centralisation during the years 1954–68 (Marsden & Steward, 1976). Reports from the GDVPTR in the US on a regional basis (Kramer *et al.*, 1984) and on a national basis in Great Britain by the Childhood Cancer Research Group in Oxford (Stiller, 1987) have shown clear benefits from such centralisation of care for both leukaemia and solid tumours.

The survival rates reported here are those actually observed in a whole population over a 15 year period during which time there have been continuing advances in our understanding and management of the problems of childhood cancer. They do not represent the optimum survival which could have been achieved either during this period or which should now be possible. Most reported series of survival in childhood cancer represent selected groups of patients and it is only when the optimum treatment can be given to all children within a defined area that we can be truly satisfied.

We are indebted to all of the consultant staff throughout the Northern Region who have ensured the success of the registry. We are also grateful to the DHSS, Newcastle Students Rag Committee, Tyneside Leukaemia Research Association, the North of England Cancer Research Campaign, and the North of England Children's Cancer Research Fund for financial support of the Registry. Mrs Pat Bagnall, Mrs Elaine Boston, Miss Shirley Brailsford, Mrs Marillyn Stewart and Mrs Lorna More have been secretaries to the Registry.

### References

BIRCH, J.M., MARSDEN, H.B. & SWINDELL, R. (1979). Acute lymphoid leukaemia of childhood in North-West England. *Lancet*, **ii**, 854.

BIRCH, J.M., MARSDEN, H.B. & SWINDELL, R. (1980). Incidence of malignant disease in childhood: A 24 year review of the Manchester Children's Tumour Registry data. *Br. J. Cancer*, **42**, 215.

BIRCH, J.M., SWINDELL, R., MARSDEN, H.B. & MORRIS-JONES, P.H. (1981). Childhood leukaemia in North-West England 1954–1977: Epidemiology incidence and survival. *Br. J. Cancer*, **43**, 324.

BLACK, D. (1984). *Investigation of possible increased incidence of cancer in West Cumbria*. HMSO: London.

BMDP (1983). *Statistical Software Manual*. Dixon, W.J. (ed). University of California Press, Berkeley, California.

CRAFT, A.W., OPENSHAW, S. & BIRCH, J.M. (1985). Childhood cancer in the Northern Region, 1968–1982: Incidence in small geographical areas. *J. Epidem. Comm. Health*, **39**, 53.

EDITORIAL (1986). Rare cancers and specialist centres. *Br. Med. J.*, **292**, 641.

EVANS, A.E., D'ANGIO, G.J. & RANDOLPH, J. (1971). A proposed staging for children with neuroblastoma: Children's Cancer Study Group A. *Cancer*, **27**, 374.

KRAMER, S., MEADOWS, A.T., JARRETT, P. & EVANS, A.E. (1983). Incidence of childhood cancer: Experiences of a decade in a population-based registry. *J. Natl Cancer Inst.*, **70**, 49.

KRAMER, S., MEADOWS, A.T., PASTORE, G., JARRETT, P. & BRUCE, D. (1984). Influence of place of treatment on diagnosis, treatment and survival in three pediatric solid tumors. *J. Clin. Oncol.*, **8**, 917.

LECK, I., BIRCH, J.M., MARSDEN, H.B. & STEWARD, J.K. (1976). Methods of classifying and ascertaining children's tumours. *Br. J. Cancer*, **34**, 69.

MARSDEN, H.B. & LAWLER, W. (1980). Bone metastasizing renal tumours of childhood. Histopathological and clinical review of 38 cases. *Virchows Arch.* (Pathol. Anatom.), **387**, 341.

MARSDEN, H.B. & STEWARD, J.K. (1976). *Tumours in Children*. Springer-Verlag: New York.

OPCS. Mid Year Population Estimates. OPCS Monitors 1968–1985.

PEARSON, A.D.J., CRAFT, A.W., PERRY, R.H., KALBAG, R.M. & EVANS, R.G.B. (1983). Four primary tumours. *Cancer*, **52**, 2363.

STILLER, C.A. & DRAPER, G.J. (1982). Trends in childhood leukaemia in Britain 1968–1978. *Br. J. Cancer*, **45**, 543.

STILLER, C.A. (1987). Increased centralisation of treatment and improvements in survival rates for cancer. *Arch. Dis. Child.* (In press).

VAN STEENSEN-MOLL, H.A., VALKENBURG, H.A. & VAN ZANEN, G.E. (1983). Incidence of childhood leukaemia in the Netherlands (1973–1980). *Br. J. Cancer*, **47**, 471.